# Chagasic Cardiomyopathy in Immigrants from Latin America to Spain 

**DOI:** 10.3201/eid1504.080938

**Published:** 2009-04

**Authors:** Ana Pérez de Ayala, José-Antonio Pérez-Molina, Francesca Norman, Rogelio López-Vélez

**Affiliations:** Ramón y Cajal Hospital, Madrid, Spain

**Keywords:** Chagas disease, cardiomyopathy, Trypanosoma cruzi, immigrants, Spain, letter

**To the Editor:** An estimated 8 million persons in 21 countries in the Western Hemisphere are infected by *Trypanosoma cruzi*, the cause of Chagas disease. The global infection rate is 1.4% (*1*) and varies by geographic area from 0.1% to 45.4% (*2*). After infection, organ involvement, predominantly cardiac disease, will develop in 20%–30% after 10–30 years (*3*).

Worldwide, Spain is second to the United States in having the largest number of immigrants from Latin America (*4*). In 2008, immigrants accounted for 11.3% of the population in Spain. A total of 1,607,699 were from *T*. *cruzi*–endemic areas; of these, 239,942 are from Bolivia (*5*), the country with the highest prevalence of *T*. *cruzi* infection (*2*).

Imported Chagas disease may emerge in Europe. Chronic Chagas disease was diagnosed in 120 patients during 2003–2008 at the Tropical Medicine Unit, Ramón y Cajal Hospital, in Madrid, Spain. Of these patients, 22.5% had cardiac involvement and 95.8% were from Bolivia. Similar data have been observed in other cities in Spain and Europe (*6*,*7*).

Successful control programs for Chagas disease have been conducted in Latin America in recent years. However, because this disease may have a latency period of many years, infection in immigrants may vary depending on background prevalence in the country of origin.

To calculate Chagas disease prevalence in Latin American immigrants, we considered different values. The number of registered immigrants in Spain according to country of origin for 2007 (*5*) and infection prevalence rates in blood donors for different disease-endemic countries (1993–2002) (*2*) were recorded. The lowest and highest prevalence rates for each country were applied to the number of immigrants from that country living in Spain. Thus, estimates were obtained for the number of potentially infected immigrants for each country of origin. From these figures, a range for the total number of potentially infected immigrants was calculated. Taking into account that all blood donors, but only 80.2% of registered immigrants in Spain (*5*), are adults, we applied an age-correction factor of 80.2% to these figures (multiplying 80.2% by the total) (Table).

**Table:** Immigrants in Spain from Chagas disease–endemic countries in South America potentially infected with *Trypanosoma cruzi*, 1993–2002*

Characteristic	No. immigrants, 2007†	Seroprevalence in blood donors, %‡	Potential no. infected immigrants§
Country			
Ecuador	420,110	0.1–0.2	420–840
Colombia	280,705	0.1–1.2	280–3,368
Bolivia	239,942	9.9–45.4	23,754–108,933
Argentina	145,315	4.4–5.5	6,393–7,992
Peru	120,272	0.1–0.2	120–240
Brazil	115,390	0.6–0.7	692–807
Venezuela	57,679	0.6–1.3	346–749
Paraguay	66,710	2.8–4.7	1,615–3,135
Chile	45,515	0.4–1.2	182–546
Uruguay	49,970	0.4–0.6	199–299
Total	1,541,608		36,567–122,232
No. adults¶	1,236,369		29,485–98,030
Estimated no. chagasic cardiomyopathies			5,897–29,409

*Infection determined on the basis of seroprevalence data from blood donors. †Data obtained from the Instituto Nacional de Estadística (*5*). ‡Data obtained from Schmunis and Cruz (*2*). §Calculated by applying seroprevalence data for blood donors in countries endemic for Chagas disease to no. immigrants from each of these countries living in Spain. ¶A correction factor for age was applied (80.2% of immigrants in Spain are adults).

Two possible scenarios were then defined to estimate the number of chagasic cardiomyopathies that may arise in the immigrant population. For the best-case scenario, the lowest calculated number of potentially infected immigrants (29,485) was used with the lowest rate of progression to cardiac involvement (20%). For the worst-case scenario, the highest number of infections (98,030) was calculated and used with a 30% risk of progression to cardiac disease. On the basis of these estimates, 5,897–29,409 cases of chagasic cardiomyopathy may be diagnosed in the near future in Spain.

Information on the prevalence of *T*. *cruzi* infection has changed over time, and the immigrant populations may not be a representative group from disease-endemic areas. Thus, extrapolating these figures to the current population in Spain may pose some problems. However, prevalence data in Bolivia (9.9% in 2001) (*2*), the country with the highest rates of infection, are consistent with data from Spain (2005–2006), which reported a seroprevalence rate of 10.2% in blood donors from Bolivia (*8*).

In recent years, vector control programs in Chagas disease–endemic countries have influenced infection rates. However, most adult immigrants became infected during their childhood, particularly in Bolivia, before any vector control programs were started. Thus, estimated infection rates in adults should not be greatly biased. In a recent study in Spain, the average age of patients with Chagas disease who came to clinics was 35 years (*6*), a finding similar to that seen at our unit. At this age, one might expect infected patients to show cardiac involvement caused by Chagas disease, as well as other manifestations such as megaesophagus and megacolon (*3*).

Screening for Chagas disease should be recommended to all Latin American migrants, especially those from Bolivia. This screening would enable early treatment for persons in the chronic asymptomatic phase or those with mild cardiac involvement, persons for whom treatment has been recommended (*9*).

Current legislation in Spain makes screening all at-risk blood donors mandatory (*10*). However, screening of pregnant women from Chagas disease–endemic countries is not compulsory, although 46.8% of immigrants in Spain are female and birth rates in this group are higher than the national average for Spain (*5*). Detection of antibodies to *T*. *cruzi* during pregnancy would also be a useful public health strategy because it would enable early specific treatment of affected newborns. Screening of blood or organ donors would also be necessary in countries where there is no transmission by vectors.

*T*. *cruzi* infection may become a public health problem in countries in Europe that receive immigrants from disease-endemic areas. Thus, chagasic cardiomyopathy may soon have a serious effect on public health in Spain.
